# A self-assembled implantable microtubular pacemaker for wireless cardiac electrotherapy

**DOI:** 10.1126/sciadv.adj0540

**Published:** 2023-10-18

**Authors:** Shaolei Wang, Qingyu Cui, Parinaz Abiri, Mehrdad Roustaei, Enbo Zhu, Yan-Ruide Li, Kaidong Wang, Sandra Duarte, Lili Yang, Ramin Ebrahimi, Malcolm Bersohn, Jun Chen, Tzung K. Hsiai

**Affiliations:** ^1^ Department of Bioengineering, University of California Los Angeles, Los Angeles, CA 90095, USA.; ^2^ Division of Cardiology, Department of Medicine, David Geffen School of Medicine, University of California Los Angeles, Los Angeles, CA 90095, USA.; ^3^ Department of Microbiology, Immunology and Molecular Genetics, University of California Los Angeles, Los Angeles, CA 90095, USA.; ^4^ Department of Medicine, Great Los Angeles VA Healthcare System, Los Angeles, CA 90073, USA.

## Abstract

The current cardiac pacemakers are battery dependent, and the pacing leads are prone to introduce valve damage and infection, plus a complete pacemaker retrieval is needed for battery replacement. Despite the reported wireless bioelectronics to pace the epicardium, open-chest surgery (thoracotomy) is required to implant the device, and the procedure is invasive, requiring prolonged wound healing and health care burden. We hereby demonstrate a fully biocompatible wireless microelectronics with a self-assembled design that can be rolled into a lightweight microtubular pacemaker for intravascular implantation and pacing. The radio frequency was used to transfer energy to the microtubular pacemaker for electrical stimulation. We show that this pacemaker provides effective pacing to restore cardiac contraction from a nonbeating heart and have the capacity to perform overdrive pacing to augment blood circulation in an anesthetized pig model. Thus, this microtubular pacemaker paves the way for the minimally invasive implantation of leadless and battery-free microelectronics.

## Teaser

A biocompatible, battery-free microtubular pacemaker revolutionizes cardiac pacing.

## INTRODUCTION

Microstimulators are widely implanted cardiac, gastric, neural, and urological devices to sustain life. In the case of cardiac pacemakers, more than half a million U.S. patients have implantable pacemakers, with the prevalence of this procedure rising from 0.4 per 1000 among persons ages 18 to 64 to 26 per 1000 among those ages 75 or order (*1*). However, pacing leads (electrodes) are prone to dislodge, fracture, and develop insulation defects as a substrate for biofilm formation (*2*–*4*). The incidence of pacing lead complications reaches ∼11%, and the rate of pacemaker casing–related complications, including infection, device erosion into the tissue, and hematoma, is estimated to be 8% at 5 years (*3*). Thus, developing the next generation of pacemakers that are both leadless and battery-free and can be implanted with a minimally invasive procedure remains an unmet biomedical engineering challenge.

To this end, leadless pacemakers have been developed to address the complications from pacing leads. Medtronic released the first U.S. Food and Drug Administration (FDA)–approved leadless cardiac stimulation device with a minimally invasive delivery to the right ventricle (*5*, *6*). However, the integrated battery introduces new clinical challenges such as device implantation, perforation through the myocardium, and dislodgement, and the current device is limited to single-chamber pacing (*7*). To ameliorate these complications, we sought to demonstrate a battery-free and leadless pacemaker that can be intravascularly deployed and implanted via a minimal invasive procedure.

Recently, flexible and self-powered devices using different energy harvesting mechanisms have been demonstrated for their battery-free operation mode (*8*–*15*). Because of the suboptimal operational stability and output power, self-powered pacing devices have remained at the investigational stage. Alternatively, wireless power transmission, including magnetic induction, radio frequency (RF), and ultrasound, represents a viable strategy to charge the pacemakers in vivo (*16*–*21*). While the optimal coil design for sufficient power transfer efficiency can be developed for the implantable electronics, open-chest surgery (thoracotomy) is required to implant the bioelectronic patches onto the epicardium (surface of the heart) (*22*–*25*). The intrapericardial method represents a minimally invasive approach for delivering soft materials to the heart, which also necessitates high flexibility in the implantable devices (*26*, *27*). Whether patients may have long-term pericardial inflammation and migration of the devices remains to be determined for clinical translation. In this context, developing a miniaturized wireless bioelectronic device for minimally invasive implantation continues to be the focus of device research (*28*, *29*).

Advances in vascular catheter–based deployment allow for implantation of biomedical devices such as arterial stents and bioprosthetic valves. Through a percutaneous incision to the femoral or radial arteries, catheter-based deployment obviates the need for thoracotomy for cardiac bypass surgery or valve replacement (*30*–*32*) and shortens recovery times and hospital stay (*33*–*36*). Recently, artificial stent and electric blood vessels enable a small and implantable endovascular device for targeted control for localized therapy (*37*, *38*). However, the primary challenge resides in the development of battery-free and implantable pacemakers with miniaturized electronics for efficient power transfer and minimal power absorption over an anatomically wireless range (*18*, *39*–*41*).

To this end, we established a self-assembled implantable microtubular pacemaker that is lightweight and wireless, providing high electrical output and operational stability for intravascular myocardial pacing and mechanical coupling. This microtubular pacemaker is designed to reduce the mechanical burden due to device fixation to the myocardium and pacing lead–related medical complications. A wireless RF module is embedded in a thin flexible polyimide membrane for receiving power transfer from the external transmitter. The polyimide membrane is selectively encapsulated with an elastomer layer to insulate the circuits. This encapsulation provides the adhesive property for the rolling self-assembly process into a microtubular device for intravascular deployment and implantation. To optimize power transfer efficiency to the microtubular pacemaker, we designed a portable RF power transmitter that enables the DC pulse delivery up to 5 V to the bipolar electrode (anode and cathode) for electrical stimulation. Following implantation to the anterior cardiac vein (ACV), we demonstrate that the microtubular pacemaker is able to reenergize the nonbeating heart; specifically, we recorded the cardiac electrocardiogram (ECG; with the pacing spike followed by QRS complex and T wave) to restore myocardial contraction, and we performed overdrive pacing to increase the heart rate and blood circulation to the hindlimb of an anesthetized pig. Thus, we demonstrate a self-assembled microtubular pacemaker for wireless and leadless myocardial stimulation.

## RESULTS

### Design of the self-assembled microtubular pacemaker

**Fig. 1.**
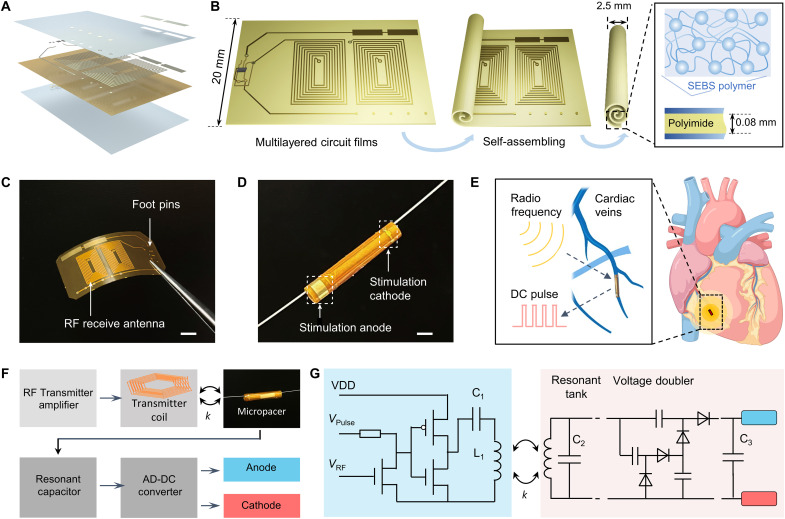
The implantable microtubular pacemaker for wireless pacing. (**A**) Multilayered circuit film for the microtubular pacemaker includes the following layers, arranged from bottom to top: SEBS encapsulation, polyimide film, circuits and components, SEBS encapsulation, and gold stimulating electrodes. (**B**) A schematic diagram of the microtubular pacemaker is fabricated by a self-assembling process, which benefits from the self-adhesiveness of SEBS polymer layer. (**C**) An optical image of an f-PCB film, which shows the two RF receiver antenna and foot pins for microcomponents. Scale bar, 5 mm. (**D**) An optical image reveals a guide wire through the microtubular pacemaker. The stimulation cathodes and anode are shown in the white dashed box. Scale bar, 2 mm. (**E**) A schematic illustration highlights the implantation of the microtubular pacemaker to the cardiac anterior vein. The microtubular pacemaker receives the RF wirelessly and generates DC pulses to stimulate the heart. (**F**) An operational diagram illustrates the wireless intravascular pacing system, where the external transmitter device generates inductive power transfer to the receiver coils of the pacemaker for electrical stimulation. (**G**) The circuit diagram of the portable RF transmitter (light blue background) and the implantable microtubular pacemaker (light orange background). VDD, Voltage Drain to Drain.

The design of the self-assembled microtubular electronics was tailored to cardiovascular anatomy and electrophysiology for wireless and battery-free stimulation. The conceptual framework is illustrated to enable intravascular implantation (Fig. 1, A and B). The flexible printed circuit board (f-PCB) membrane allowed for a thickness of 80 μm, consisting of three modules: (i) A pair of antennas receives the RF energy from an external transmitter (Fig. 1C), (ii) the rectifier circuit with a voltage doubler unit effectively converts the AC waveforms to DC pulses (foot pins for the electronic components in Fig. 1C), and (iii) the anode and cathode electrodes deliver the DC pulses for myocardial stimulation (Fig. 1D). The smaller electrodes for the cathode were designed to maintain an optimal current density of electron streams, thus reducing power consumption needed for cardiac stimulation.

To encapsulate the f-PCB film, we encapsulated a layer of styrene-ethylene-butylene-styrene (SEBS) polymer by mask-spraying method (fig. S1). Because of the excellent resistance to water, the SEBS layer can insulate the f-PCB from corrosion in the electrolyte-rich environment, thus providing a long-term operational stability for the implantable device (*42*–*44*). Furthermore, this thin SEBS encapsulation provides a self-adhesive property for the self-assembly rolling process to form a stable microtubular structure (*45*). The stimulating electrodes were sputtered with a layer of gold to provide electrochemical stability in the electrolyte-rich environment (*46*–*49*). Next, this integrated circuit membrane was rolled into a microtube to facilitate intravascular implantation into the ACV (Fig. 1D). The complete fabrication process flow is illustrated in fig. S1. Notably, a guidewire through the hollow microtubular structure facilitates the intravascular deployment, similar to the catheter-based deployment of cardiac stents (Fig. 1D). In addition, with a hoop design on the end of the microtubular pacemaker, it can be retrieved by a catheter, as shown in fig. S2.

To effectively control the power transfer of the RF energy to the microtubular electronics, we developed a portable transmitter (fig. S3). The portable RF transmitter consisted of a class D push-pull amplifier and a microcontrol unit (MCU) to optimize the coupling of inductive power to the receiver coils. The integrated circuit of the microtubular electronics enabled the resonant capacitor to receive the AC waveforms from the external transmitter coil, and the AC waveforms were converted to the rectified DC pulses and delivered to the anode and cathode electrodes (Fig. 1, E and F). In the integrated stimulation system (Fig. 1G), the portable transmitter circuit provided a monopolar pulse voltage ranging from 0 to 5 V to control the duration and period of the RF waveform group, and the RF signal (*V*_RF_) was applied to the gate of an n-type metal oxide–semiconductor field-effect transistor and then was fed to the class D RF amplifier (fig. S3). The inductance L_1_ of the transmitter coil was resonantly coupled with a capacitor C_1_ in series, thereby enabling the alternative and efficient induction of magnetic field to the receiver coils, and the inductance L_2_ of the receiver antenna was resonantly coupled with a capacitor C_2_. Rectification of the RF AC signals to DC pulses was made possible by a voltage doubler unit. The DC energy was stored in the capacitor C_3_, which filtered the overlapped ripples before delivering the DC pulses to the anode and cathode electrodes.

### Optimizing power transfer efficiency via magnetic field simulation

**Fig. 2.**
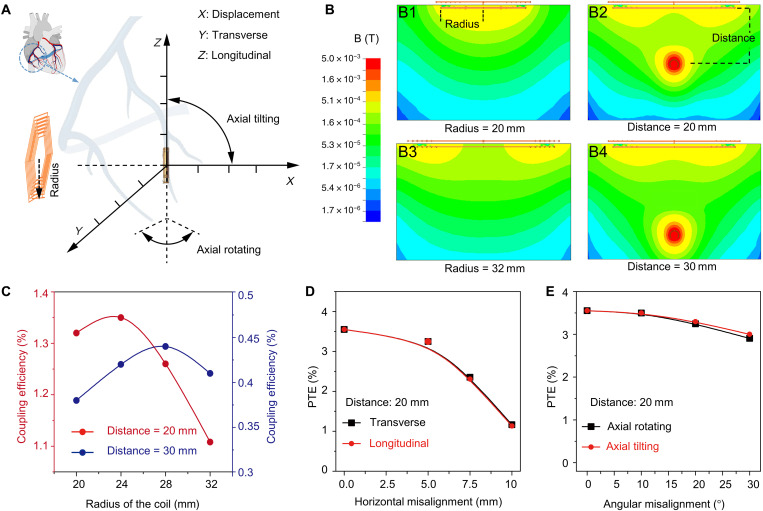
Simulation of coupling efficiency for inductive power transferring. (**A**) A schematic diagram reveals the displacement, misalignment (transverse direction and longitudinal direction), and angular misalignment that may affect the PTE. (**B**) (B1 and B3) Magnetic fields were simulated at various radii of the transmitter coil (*r* = 20 to 32 mm). (B2 and B4) The coupling efficiency was dependent on the displacement ranging from 20 to 30 mm. (**C**) The coupling efficiency as a function of radius of the coil was compared between the distance of 20 and 30 mm. (**D**) There is a correlation in PTE as a function of horizontal misalignment from 0 to 10 mm between the transverse and longitudinal direction. (**E**) There is also a correlation in PTE as a function of angular misalignment from 0^o^ to 30^o^ between axial rotation and tilting.

To determine the optimal power transferring efficiency (PTE), we simulated the magnitude of the magnetic field radiated from the transmitter coil to the receiver coil using ANSYS software (Fig. 2A). The maximum strength of the magnetic field occurred in the region most proximal to the transmitter coil. As the displacement increased, the magnetic field for the central point declined proportionally to the reciprocal of the radius. In the normal direction, the magnetic field was highly related to the radius of the coil and the displacement to the transmitter coil plane. While a small transmitter radius would result in an insufficient magnetic field to the out-of-plane point, a large radius would lead to the out-of-plane point too far to achieve sufficient magnetic field. As illustrated in Fig. 2 (B1 to B4), the three-dimensional (3D) model for the transmitter coil in ANSYS used copper wires that were configurated into a two-layer hexagon with a span of 1.0 cm and six turns. As the outer radius increased from 20 to 32 mm, the distribution of the magnetic field was enlarged from 20 to 30 mm beyond the transmitter coil plane (fig. S5). At a displacement of 20 mm, the maximal coupling efficiency was achieved at the outer radius of ~24 mm; and at a displacement of 30 mm, the maximal efficiency was achieved at the radius of ~28 mm (Fig. 2C). Furthermore, the coupling efficiency at a displacement of 20 mm increased by more than twofold as compared to that of 30 mm, from 0.44 to 1.35%. Therefore, the magnetic field simulation enhanced the design of the hexagonal transmitter coil to optimize the coupling efficiency.

Myocardial contraction generates a periodical misalignment around the original position during a cardiac cycle. For this reason, alignment of the transmitter and receiver coil influences the efficiency in inductive powering transferring. We thus investigated the impact of misalignment on our PTE. A horizontal misalignment by 10 mm in transverse or longitudinal direction resulted in a reduction in PTE by >50% (Fig. 2D and fig. S6), therefore demonstrating that the strength of the magnetic field in the region off the normal direction to the transmitter coil decayed rapidly. However, angular misalignment via axial rotation or lateral rotation to a 30° resulted in a small reduction in PTE by ~10% (Fig. 2E and fig. S7).

### Characterizations of the microtubular electronics

**Fig. 3.**
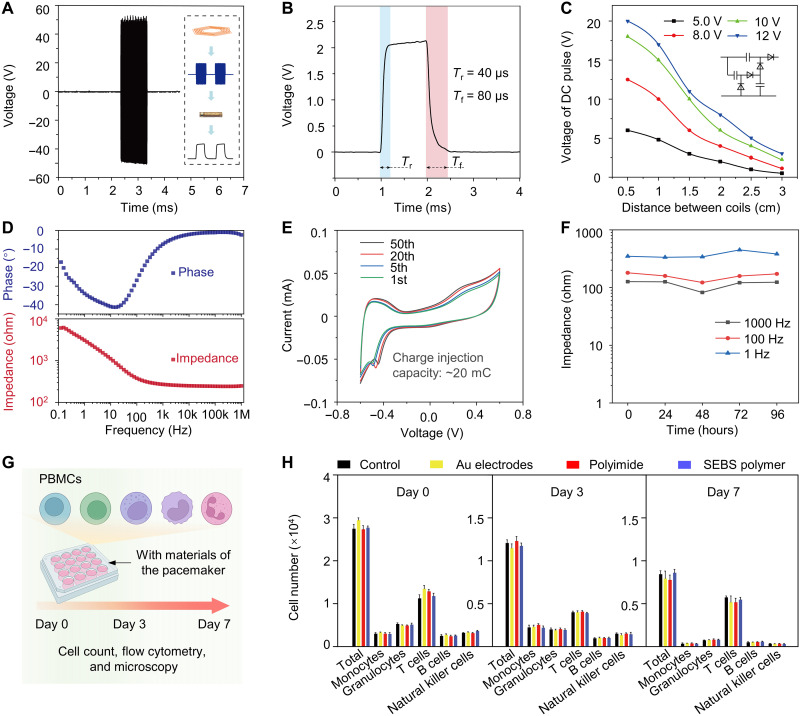
Electrical characterizations and cytotoxicity assay of the microtubular pacemaker. (**A**) In the transmitter unit, the RF amplifier increased the amplitude of *V*_RF_ signals to the transmitter coil. (**B**) A single stimulation pulse signal delivered a rapid rise and decay in voltage to the anode and cathode. (**C**) The rectification was achieved via a voltage doubler to deliver the DC pulses to the anode and cathode. The voltage of the DC pulse was dependent on the strength of the magnetic field, which decayed rapidly as the displacement increased from 0 to 3.0 cm. (**D**) The representative impedance and phase-angle spectra of the stimulating electrodes that were sputtered with the gold (Au) layer. (**E**) Cyclic voltammograms of a representative stimulation electrode at the 1st, 5th, 20th, and 50th cycles in PBS solution. (**F**) Change in impedance at the frequency of 1, 100, and 1000 Hz of the stimulation electrode over 96-hour immersion in the PBS solution. (**G**) The experiment design for the biocompatibility assay. (From left to right: B cell, T cell, natural killer cell, monocyte, and granulocyte). (**H**) A plot of various immune cell numbers over time indicates that the device imparted minimal cytotoxic effects on activation of various immune cell populations.

**Fig. 4.**
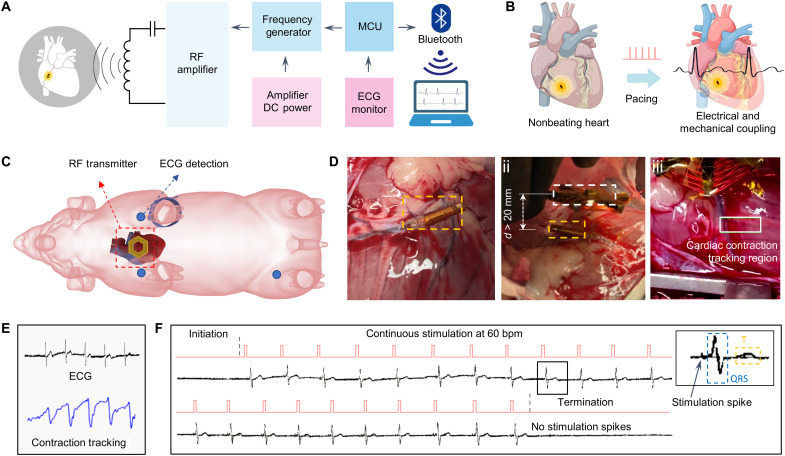
Intravascular implantation of the microtubular pacemaker to restore cardiac conduction and myocardial contraction. (**A**) A schematic illustrates the portable system combining an RF transmitter and an MCU to achieve wireless power delivery and ECG data acquisition. (**B**) Intravascular implantation of the microtubular pacemaker to the anterior vein of a nonbeating heart. Induction of transmitter coil for magnetic interaction with the receiver coil. (**C**) RF transmitter coil is placed above the epicardium where the microtubular pacemaker is implanted to the ACV in an anesthetized pig. The ECG pads were connected to the left upper limb, right upper limb, and left lower limb. (**D**) (Di) Implantation of the microtubular pacemaker in the ACV. (Dii) Displacement between external transmitter (white dashed box) and the microtubular pacemaker (orange dashed box) was greater than 20 mm for inductive power transferring. (Diii) Myocardial contraction was recorded by the video tracking of an epicardial region of the left ventricle. The contraction region was labeled, and the moving coordinates around the center of the region were calibrated to synchronize with the ECG. (**E**) Video tracking of myocardial contraction in response to electrical stimulation. (**F**) ECG rhythm strip reveals the initiation of electrical stimulation in the nonbeating heart at 60 bpm as evidenced by the consistent pacing spikes, followed by QRS for myocardial activation and T waves for repolarizations (inset). Removal of the RF transmitter coil terminated the stimulation spikes.

Before implanting the microtubular pacemaker for cardiac pacing, we characterized the strength of our inductively powered system via in vitro testing. The initiation and termination of the RF pulses were coupled with rise and fall of the DC pulses (Fig. 3, A and B). Two-circuit architecture was used to compare RF/AC-DC conversion with the voltage doubler versus full bridge rectifier (Fig. 3C and fig. S8). In the transmitter unit, the RF amplifier gradually increased the transmission power voltage from 5.0 to 12.0 V to the receiver coil. In the stimulation unit, the voltage of the DC pulse was rectified and delivered to the anode and cathode. As expected, as the displacement between the transmitter coil and receiver coil increased from 0.5 to 3.0 cm, the coupling efficiency declined, resulting in a decrease in the voltage of DC pulse (Fig. 3C). Note that by increasing the operating voltage of the transmitter, the DC pulse can be reliably maintained above 2 V, even when the distance between the transmitter and pacemaker exceeds 3 cm (fig. S9). This ensures that it fulfills the requirements for patients with greater chest-to-skin distances (*50*). The voltage doubler excited the electrons to a higher potential to double the amplitude of the RF/AC signal, leading to additional increase in the voltage of the DC pulses for cardiac stimulation. Thus, the voltage for the DC pulse generated from the microtubular pacemaker provided sufficient energy threshold to reenergize the nonbeating heart in the euthanized pig (Fig. 4).

Electrical impedance spectroscopy was performed to evaluate the electrical impedance (ohm) and phase (θ) of the stimulating electrodes over a frequency range (0.1 Hz to 1 MHz) (Fig. 3D). The impedance of the Au-sputtered stimulating electrodes maintained at ~110 ohms until the frequency was lower than 100 Hz in the phosphate-buffered saline (PBS) solution. The phase plot of the impedance spectrum revealed a phase angle close to 0° when the frequency was above 100 Hz. The electrical impedance spectroscopy performance supports the dominance of the resistance component suitable for electrophysiological simulations. In Fig. 3E, the voltammogram cycles showed a wide bandwidth of electrochemical stability of the Au-sputtered stimulating electrodes from a linear voltage sweep in PBS (−0.6 to 0.6 V). The voltammogram demonstrates a reliable electrochemical stability under the physiological conditions (*51*). In addition, the microtubular electronics was assessed for long-term stability (Fig. 3F), and the impedance magnitude of an individual stimulating electrode remained stable over the 96-hour immersion in PBS.

### Biocompatibility testing of the microtubular electronics

To evaluate the biocompatibility of the materials, an in vitro incubation assay was performed using the human peripheral blood mononuclear cells (PBMCs) as a well-accepted immune cell population for investigating biocompatibility and inflammatory responses to various materials (*52*–*54*). By virtue of PBMC’s heterogeneous population, consisting of granulocytes, monocytes, and lymphocytes (natural killer cells, T cells, and B cells), these cells actively participate in both inflammatory and immune responses (*55*, *56*). We hereby incubated PBMCs with the materials used in our microtubular pacemaker, including the Au electrodes, polyimide, and SEBS polymer. Flow cytometry [fluorescence-activated cell sorting (FACS)] analysis was performed to evaluate the viable cell population, including the total cells, monocytes, granulocytes, T cells, B cells, and natural killer cells (Fig. 3G). Cell counts (Fig. 3H) and FACS analyses (fig. S10) indicated the absence of cell toxicity and immune responses for up to 7 days. Microscopic examination of the cultured cells further revealed the similar cell densities in response to the materials used in the microtubular pacemaker (fig. S11), consistent with those of FACS analysis. To further demonstrate hemocompatibility, we conducted a series of ex vivo experiments to observe thrombotic responses. As shown in fig. S12, in the absence of heparin, the blood forms clots in both test and control groups as evinced by the nonhomogeneous appearance. In the presence of heparinization, the blood appears homogeneous in coloration in these groups. Collectively, these experiments demonstrated the biocompatible materials used in the microtubular pacemaker.

### Intravascular pacing to restore electromechanical coupling and blood circulation

The porcine whole-heart models closely resemble human physiology and are clinically translational for in vivo studies. Here, in vivo implantation in a Yorkshire pig demonstrated how this microtubular, leadless, and battery-free pacemaker is capable of delivering electrical stimulation up to DC of 5 V. To provide continuous pulse transfer to the implanted pacemaker, we developed a portable system that combines an RF transmitter and an MCU to achieve wireless power delivery and ECG data acquisition (Fig. 4A). To acquire the ECG signal from the heart, we intravascularly implanted the microtubular pacemaker to the ACV in a Yorkshire pig (Fig. 4B) immediately after euthanasia in compliance with the Institutional Animal Care and Use Committee (IACUC). An experienced veterinarian performed a thoracotomy to provide the surgical window of the epicardium of the pig heart and to allow for dissection and isolation of one of the ACVs for intravascular implantation of the microtubular pacemaker. The stimulating electrodes of the microtubular pacemaker achieve intimate contact with the walls of the selected cardiac vein. This ensures that electrical stimulation can be effectively delivered into the heart while also preventing pacemaker slippage along the vein. ACV was chosen for its proximity to the chest wall as the ideal region for optimal inductive power transfer and for its proximity to His-Purkinje fiber (the conduction bundle) for generation of the narrow QRS complex (as opposed to the wide QRS complex associated with ventricular pacing). The ECG leads were connected to the forelimbs and the left hindlimb of the animal for real-time recording in response to inductive power transfer and myocardial stimulation (Fig. 4C).

Intravenous implantation of the microtubular pacemaker to reenergize electrical conduction and myocardial contraction was set at 1 Hz [60 beats/min (bpm)] at a pulse duration of 1 ms. The microtubular pacemaker was implanted to the cardiac anterior vein, and the RF transmitter coil was positioned at ~2.0 to 3.0 cm above the receiver coils (Fig. 4, B and C). In response to the wireless power transmission, the surface ECG recorded real-time cardiac conduction before and after the stimulation spikes (Fig. 4D). The ECG waveform was flat before stimulation, and upon inductive power transfer, the ECG demonstrated QRS complexes and T waves at 60 bpm. After removal of the transmitter coil, the ECG returned to a flat line. Figure 4Diii shows the continuous tracking of the cardiac contraction from the left ventricle epicardial region, demonstrating the electrical and mechanical coupling in response to intravascular pacing (Fig. 4F and fig. S14). Distinct ECG patterns were observed (inset), revealing the stimulation spike, followed by the narrow QRS for ventricular depolarization, and T wave for repolarization during a cardiac cycle. This cardiac stimulation was performed for >20 cycles to demonstrate electrical and contraction coupling. The myocardial contraction in response to electrical stimulation was recorded as epicardial movements (Fig. 4E and movie S1).

**Fig. 5.**
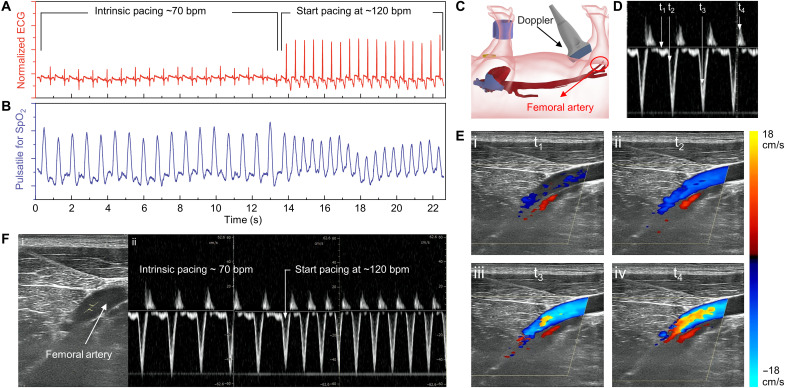
Intravascular implantation of the microtubular pacemaker to restore hemodynamic blood flow. (**A** and **B**) ECG and SpO_2_ monitoring in response to cardiac contraction from 70 to 120 bpm. (**C**) The schematic illustrates the placement of duplex ultrasound to detect blood flow from the femoral artery. (**D**) Pulsed wave Doppler detected the pulsatile arterial blood flow in response to cardiac contraction. (**E**) t_1_ to t_4_ indicate the Doppler signals at different cardiac cycles. t_1_ corresponds to diastole, t_2_ corresponds to early systole, t_3_ corresponds to mid-systole, and t_4_ corresponds to end systole. The color gradients indicate the direction and magnitude of blood velocity toward the hindlimb. (**F**) Pulsed wave Doppler detected femoral arterial blood flow in response to cardiac pacing from 70 to 120 bpm.

We further demonstrate that this electrical and mechanical coupling is able to perform overdrive pacing and increase blood circulation to the hindlimbs of the anesthetized pig. In Fig. 5 (A and B), the RF transmitter coil was able to increase the rate of power transfer to the receiver coils, resulting in continuous wireless pacing from ~70 to 120 bpm, as evidenced by the ECG acquisition. In parallel, we monitored oxygen saturation of the peripheral artery (SpO_2_) during cardiac stimulation (movie S2). In Fig. 5 (C and D), an ultrasound probe was placed over the femoral artery, and pulsed wave Doppler detected the pulsatile blood flow during different states of a cardiac cycle (movies S3 and S4). T_1_ corresponds to diastole, while t_2_ represents early systole, t_3_ represents mid-systole, and t_4_ represents end-systole.

## DISCUSSION

A self-assembled implantable microtubular pacemaker was developed for intravascular deployment and wireless pacing to restore cardiovascular function. We demonstrated the battery-free and implantable pacemakers for efficient power transfer and minimal power absorption over an anatomically wireless range (*18*, *39*–*41*). This microtubular pacemaker is promising for intravascular deployment and implantation for the miniatured size to deliver up to DC of 5 V to overcome myocardial pacing threshold.

The design of the thin f-PCB film with an SEBS encapsulation enabled a self-assembled rolling process to form the microtubular structure, thus allowing the deployment to the ACV (<3 mm). The magnetic field simulation guided an optimized circuit design to achieve a power transfer efficiency of ~1.3% at distances up to 3 cm. The stimulating electrodes of the microtubular pacemaker are well designed to contact with the endoluminal wall, and the contact area was comparable to that of the distal electrode for the commercially designed leadless pacemaker (such as Medtronic Micra) (*57*, *58*). The in vivo demonstration on a pig model has further substantiated the functionality of the wireless intravascular pacing methodology via ACV implantation.

The frequency of 1.0 MHz used for our inductive power transfer was to reduce radiation absorption by the bio-tissues and attenuation of power transfer due to the higher frequency band. The inductive power transferring at 1.0 MHz also met the Federal Communication Commission specification (section 15.209) for the use of small antennas with low energy. The specific absorption rate (SAR) was simulated to be ~1.0 × 10^−4^ W/kg (temporal average), less than the FDA-specified safety level at 10 W/kg (*56*, *58*, *59*). The hemocompatibility of the microtubular pacemaker has been demonstrated via anticoagulation to reduce the risk of acute thrombosis and immune responses.

Overall, this self-assembled microtubular pacemaker provides an innovative entry point for intravascular deployment with translational implication for cardiac, gastric, neural, and urological stimulation. The self-assembled microtubular pacemaker has the capacity to jumpstart a nonbeating heart or to perform overdrive pacing from 70 bpm at resting to 120 bpm needed during physical activity. This implementation eliminates the need for a charge storage unit in the bioelectronics and obviates the need for open-chest thoracotomy, prolonged wound healing and health care burden.

## MATERIALS AND METHODS

### Materials

All chemical reagents were commercially available, including PBS (0.01 M; pH 7.4), xylene from Sigma-Aldrich, USA. Asahi Kasei Corporation provided SEBS (SEBS-H1221) elastomer. As regard the formulation of the SEBS solution for spray coating, 5 g of SEBS-H1221 elastomer was mixed with 100 ml of xylene and stirred for 5 hours. The gold target (57 × 0.1 mm, 99.99%) for sputter coating is purchased from Ted Pella Inc. The polyimide-based flexible PCB circuit membrane was designed by the Altium Designer software and fabricated with the help of the PCBWay company, and the thickness of the membrane is about 80 μm. Fluorochrome-conjugated antibodies specific for CD45 (clone H130), CD3 (clone OKT3), CD11b (clone ICRF44), CD19 (clone HIB19), CD56 (clone HCD56), CD14 (clone HCD14) were purchased from BioLegend. Human Fc receptor blocking solution (TrueStain FcX) was purchased from BioLegend. Fixable viability dye eFluor 506 (e506) were purchased from Affymetrix eBioscience.

### Fabrication of the device

The f-PCB membranes were fabricated by a commercialized PCB manufacturer (PCBWay) based on the designs formed using Altium Designer software. The electrical components for the flexible f-PCB membrane include capacitors, diodes, and voltage doubler complementary metal oxide semiconductor chips. The stimulation electrodes of the f-PCB were sputtered with a layer of gold with a thickness of ~100 nm, which have an excellent electrochemical stability in electrolyte environment. A layer of SEBS elastomer encapsulation was coated on the f-PCB through the masked-spraying method to not only further protect the integrated circuit (IC) from electrolyte environment but also provide the adhesive property for the following self-assembled rolling process. The thin f-PCB membrane was then rolled into a microtube with a diameter of ~2.5 mm. The surface adhesiveness of the SEBS encapsulation contributes to the stable microtubular structure.

Compared to traditional circular and square coils, the hexagonal coil offers distinct advantages, including seamless splicing and flexible combination, superior antimisalignment characteristics, and a higher level of cost-effectiveness (*60*–*62*). Consequently, we have chosen to design the transmitter coil in a hexagonal shape. The transmitter coil was designed with the insulator-coated copper wires (AWG 26), resulting in a hexagonal configuration with a radius of 30 mm, an inductance of 38 μH, and a resistance of 2.6 ohms at 1.0 MHz (fig. S3). The transmitter can continuously operate for ~5 hours given a power consumption of 200 mW and 1000-mAh battery. The frequency of 1.0 MHz was used for our inductive power transfer to reduce radiation absorption by the bio-tissues and attenuation of power transfer due to the higher frequency band. The copper wire of the transmitter coil was separated between the adjacent turns, spanning around 1.0 cm for six turns on a plane to reduce the parasitic capacitance and to increase the strength of magnetic field region in the normal direction to the transmitter coil. The skin depth, the depth where the current density is just 1/*e* ~ 37% of the value at the surface, of the copper coil wires was higher at a low frequency, thus reducing the AC resistance and increasing the corresponding quality factor (*Q* value). The inductive power transferring at 1.0 MHz also met the Federal Communication Commission specification (section 15.209) for the use of small antennas with low energy. The SAR was simulated to be ~1.0 × 10^−4^ W/kg (temporal average), far below the FDA-specified safety level at 10 W/kg (*51*, *52*).

### Electromagnetic simulation

The commercial software package ANSYS HFSS (ANSYS) was used to perform electromagnetic simulation to determine the optimal PTE. The 3D model for the transmitter coil in ANSYS used the copper wires that were configurated into a two-layer hexagon with a span of 1.0 cm for six turns. The transmitter coils with an outer diameter of 20, 24, 28, 30, and 32 mm are tuned to operate at a resonant frequency of 1 MHz. The distance between transmitter coil and the microtubular pacemaker was set at 2 and 3 cm, respectively. Considering that during the in vivo application scenarios, the dynamic beating heart can cause horizontal and angular misalignments that may influence the PTE, we further simulated these situations. The horizontal misalignments of 0.0, 5.0, 7.5, and 10.0 mm were simulated at transverse and longitudinal direction, respectively. For the angular misalignments, we set 0°, 10°, 20°, and 30° as the axial rotating and tilting degree. The biological tissue parameters for SAR simulation include the dielectric constant (ɛ), electrical conductivity (σ), and density (ρ), with the following values: ɛ = 246, σ = 0.53 S/m, and ρ = 1055 kg/m^3^. For continuous pacing, we used an external coil with 12 turns and an input power of 2 W, placed directly in front of the chest model.

### Characterizations

The waveforms of the RF and stimulation DC pulse were performed with the digital storage oscilloscope (B&K PRECISION 2190D). To characterize the wireless power transmission efficiency of the microtubular electronics, the voltages of the transmitter were set at 5, 8, 10, and 12 V with the distance varied from 0.5 to 3 cm. The electrochemical characterizations were conducted under a three-electrode configuration by using the Interface 1010E potentiostat. The electrochemical stability windows of the stimulation cathodes and anode were measured by linear sweep voltammetry at a scan rate of 5 mV/s in PBS solution. Electrochemical impedance spectroscopy was measured in the frequency range from 0.1 Hz to 1 MHz with a 5-mV amplitude at the open-circuit potential.

### In vivo pig model experiments

To evaluate the feasibility of the device for cardiac pacing, we conducted ex vivo experiments on male Yorkshire pigs (*n* = 4) weighing more than 50 kg and more than 14 weeks of age. All animal experiments were conducted in accordance with protocols approved by the University of California, Los Angeles (UCLA) IACUC. The pigs were anesthetized using intramuscular ketamine and midazolam, while fentanyl was administered intravenously for pain control during surgery. To maintain surgical plane of anesthesia throughout the procedure, 1 to 3% isoflurane was administered via endotracheal tube, and the animals were mechanically ventilated. A 6F introducer sheath was percutaneously inserted using the Seldinger technique into the right or left femoral artery to monitor blood pressure. Bupivacaine was subcutaneously injected into the chest, and a midline sternal incision was made to gain access to the thorax. Rib spreaders were used to expand the incision and expose the heart. The pericardium was incised to enable access to the heart.

The animal was humanely euthanized using a combination of pentobarbital and phenytoin, administered intravenously. Subsequently, we inserted the device by creating an incision in the ACV downstream of the implant location. The intravascular pacemaker was then introduced into the vessel via the opening. To achieve optimal positioning of the wireless power transmitter, we referred to previous thoracic magnetic resonance imaging studies, which have demonstrated a mean distance of 20 mm between the intravascular pacemaker in the ACV and the subcutaneous transmitter in humans. On this basis, we placed the wireless power transmitter at a distance of 20 mm from the intravascular pacemaker. Immediately following euthanasia, pacing was initiated at a rate of 60 bpm to minimize cellular apoptosis and release of intracellular electrolytes. ECG readings were then observed and recorded to evaluate the pacing function. Video tracking of myocardial contraction was achieved by object tracking algorithms in OpenCV library.

### Flow cytometry

All flow cytometry stains were performed in PBS for 15 min at 4°. Samples were stained with the fixable viability dye e506 mixed with human Fc receptor blocking solution (TrueStain FcX), followed by PBS washing to remove e506 and blocking antibodies. Antibody staining was added to all samples at specified dilutions according to the manufacturer’s instructions. Flow cytometry was performed using a MACSQuant Analyzer 10 flow cytometer (Miltenyi Biotech), and FlowJo software version 9 was used for data analysis.

### Biocompatibility testing in human PBMCs

Healthy donor PBMCs were obtained from the UCLA CFAR Virology Core Laboratory, in accordance with federal and state regulations, without identification information. The biocompatibility of varied materials was assessed by culturing the healthy donor PBMCs in tissue culture treated nonpyrogenic polystyrene 24-well cell culture plates, using complete lymphocyte culture medium (C10 medium). RPMI 1640 supplemented with 10% (v/v) fetal bovine serum, 1% (v/v) Penicillin/Streptomycin/Glutamine, 1% (v/v) minimum essential medium nonessential amino acids, 10 mM Hepes, 1 mM sodium pyruvate, 50 mM 2-mercaptoethanol, and Normocin (100 mg/ml) was used to prepare C10 medium for all PBMC-related cultures. The PBMCs were cultured for 3 and 6 days in C10 medium, and their viability was then assessed. Viability was determined by staining the live cells with e506 viability dye. CD3^+^ cells were used to identify human T cells, CD19^+^ cells were used to identify B cells, CD3^−^CD56^+^ cells were used to identify natural killer cells, CD14^+^CD11b^+^ cells were used to identify monocytes, and CD11b^+^CD14^−^ cells were used to identify granulocytes.
