# Supplementary material for: A self-assembled implantable microtubular pacemaker for wireless cardiac electrotherapy
Source: Sci Adv. 2023 Oct 18;9(42):eadj0540. doi: 10.1126/sciadv.adj0540 (PMC10584332; doi:10.1126/sciadv.adj0540)
Supplement: Supplementary file 1 — Figs. S1 to S15 Legends for movies S1 to S4 [file sciadv.adj0540_sm.pdf]

Science Advances
AAAS

Supplementary Materials for

A self-assembled implantable microtubular pacemaker for wireless cardiac electrotherapy

Shaolei Wang *et al.*

Corresponding author: Tzung K. Hsiai, thsiai@mednet.ucla.edu

Sci. Adv. **9**, eadj0540 (2023)
DOI: 10.1126/sciadv.adj0540

The PDF file includes:

Figs. S1 to S15
Legends for movies S1 to S4

Other Supplementary Material for this manuscript includes the following:

Movies S1 to S4

Supplementary Figures

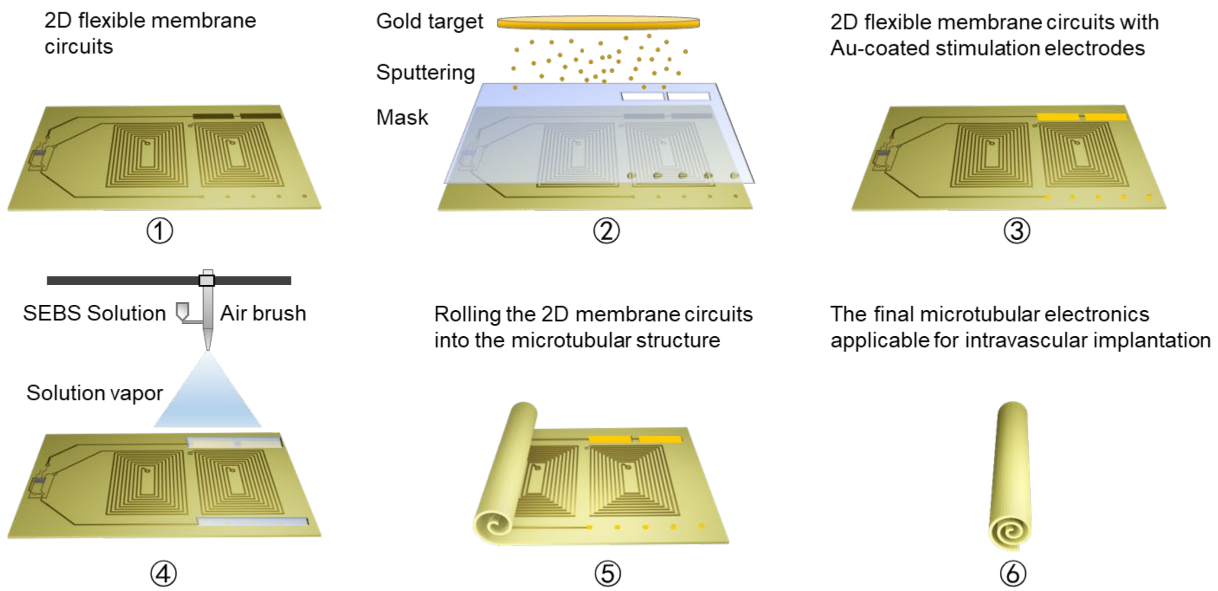

Fig. S1. **Schematic fabrication processes of the microtubular pacemaker.** After a series of coating on the flexible circuits, a rolling process is applied on the 2D flexible circuit membrane to form a microtubular structure.

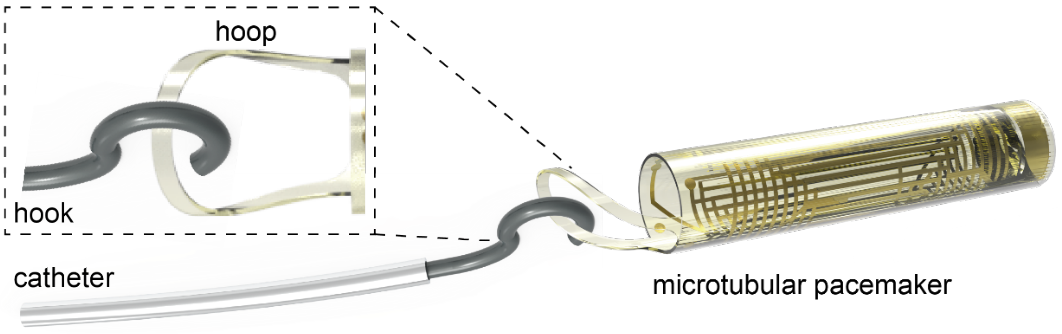

Fig. S2. **Schematic figure explains the retrieval structure.** The schematic figure illustrates that the microtubular pacemaker has a hoop structure at its end, allowing for retrieval using a catheter.

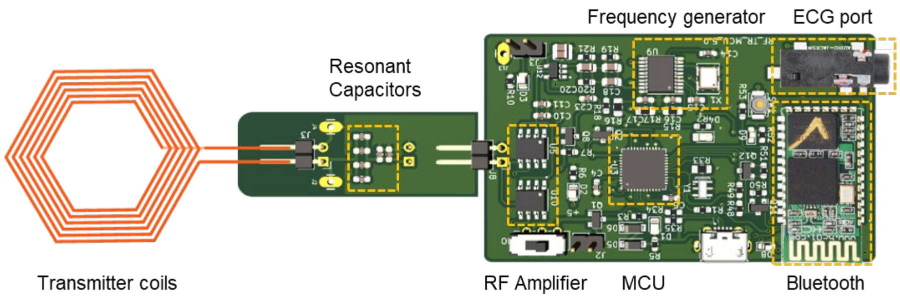

Fig. S3. **A portable module with RF transmitter and ECG porting.** The portable transmitter coil was designed to optimize inductive power transferring to the receiver.

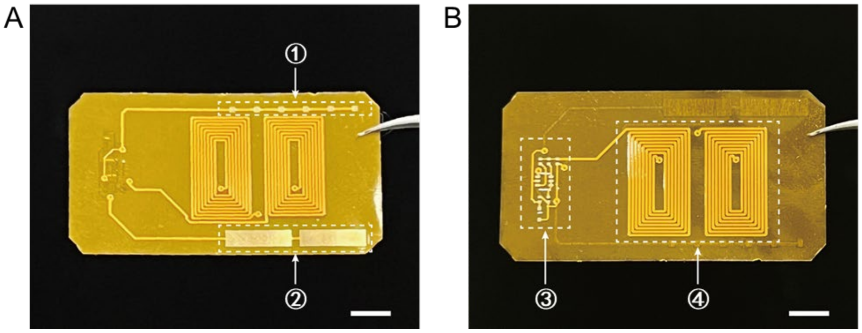

Fig. S4. **The integrated polyimide-based circuit membrane for intravascular implantation.** (**A**) On the outer side, the cathode consisted of 6 small gold-coated electrode pads (white dotted area 1), whereas the anode consisted of a pair of electrode pads aligned on the opposite border (white dotted area 2). Scale bar: 5mm. (**B**) On the inner side of the Polyimide membrane, the resonant, storage capacitors, rectifier, and regulator diodes were packaged in the “foot-pins of the components” (white dotted area 3). The integrated polyimide-base circuit membrane enabled inductive power transfer to a pair of receiver antenna (white dotted area 4). Scale bar: 5mm.

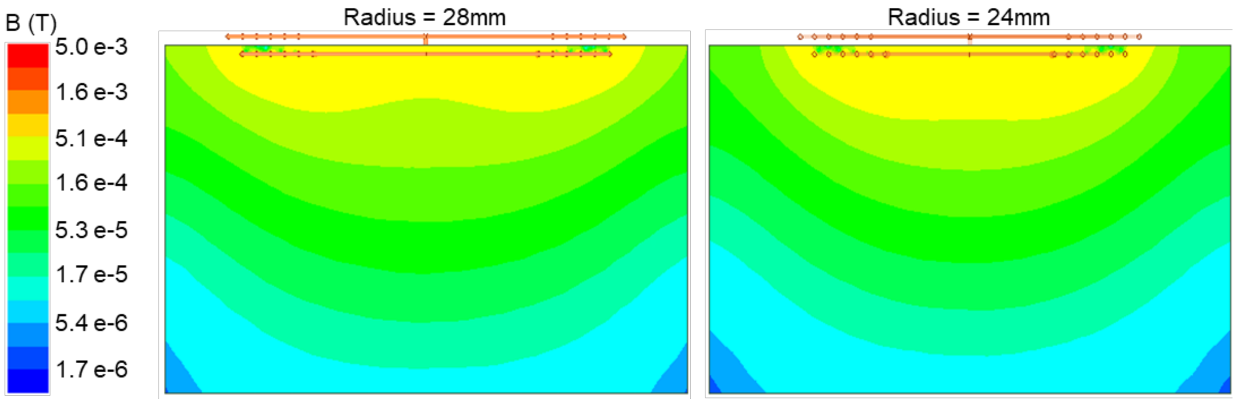

Fig. S5. Simulation results for the magnetic fields of transmitter coil. Magnetic fields were simulated at 20 mm and 32 mm radius of the transmitter coil.

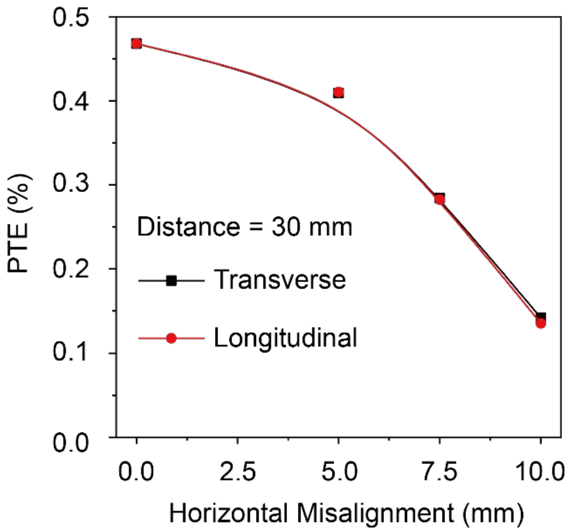

Fig. S6. The correlation between horizontal misalignment and power transfer efficiency. Power transfer efficiency at a distance of 30mm as the horizontal misalignment increased from 0 mm to 10 mm in both the transverse and longitudinal direction.

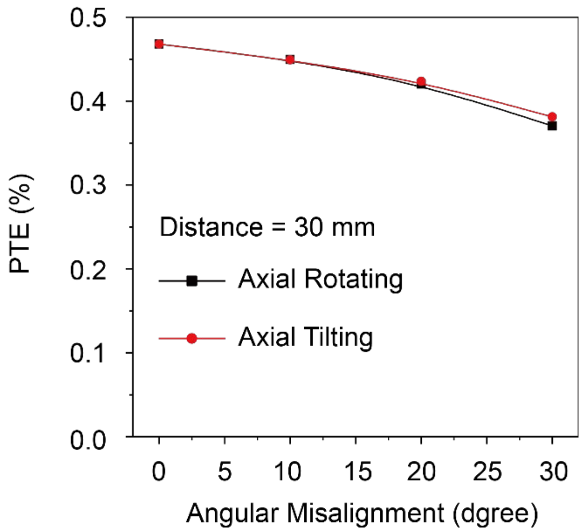

Fig. S7. The correlation between angular misalignment and power transfer efficiency. Power transfer efficiency (PTE) at a distance of 30mm as the angular misalignment increased from 0 degree to 30 degree in both the rotating and tilting form.

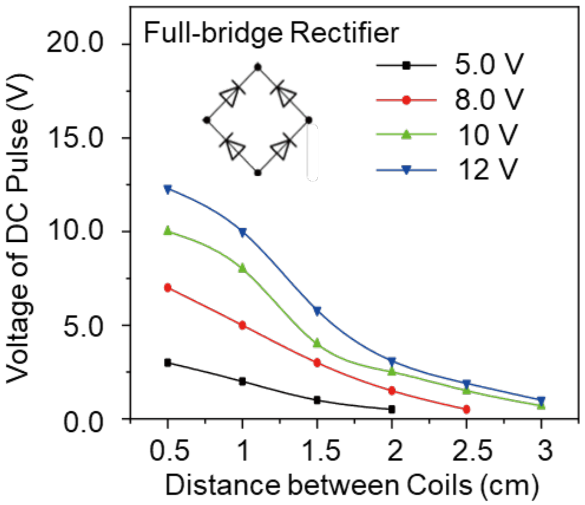

Fig. S8. Electrical output of the full bridge rectifier. The voltage of the DC pulse was dependent on the strength of the magnetic field, which decayed rapidly as the displacement increased from 0 to 3.0 cm.

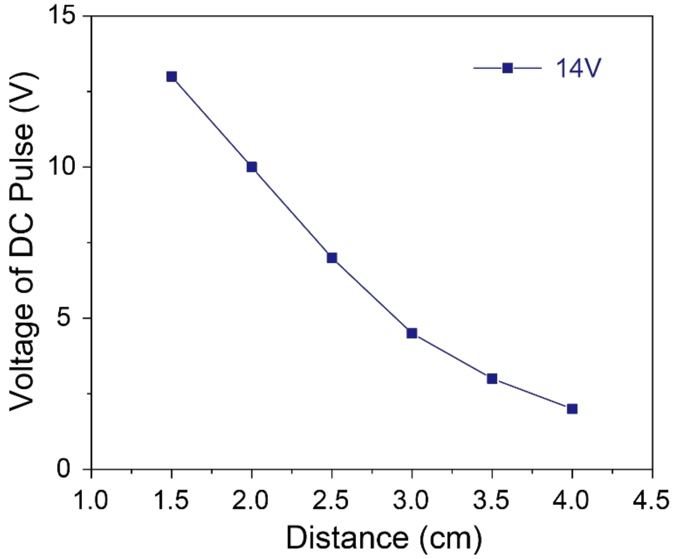

Fig. S9. Electrical output of the pacemaker. The relationship between voltage of the DC pulse and RF transfer distance when the operating voltage (VDD) is set at 14 V.

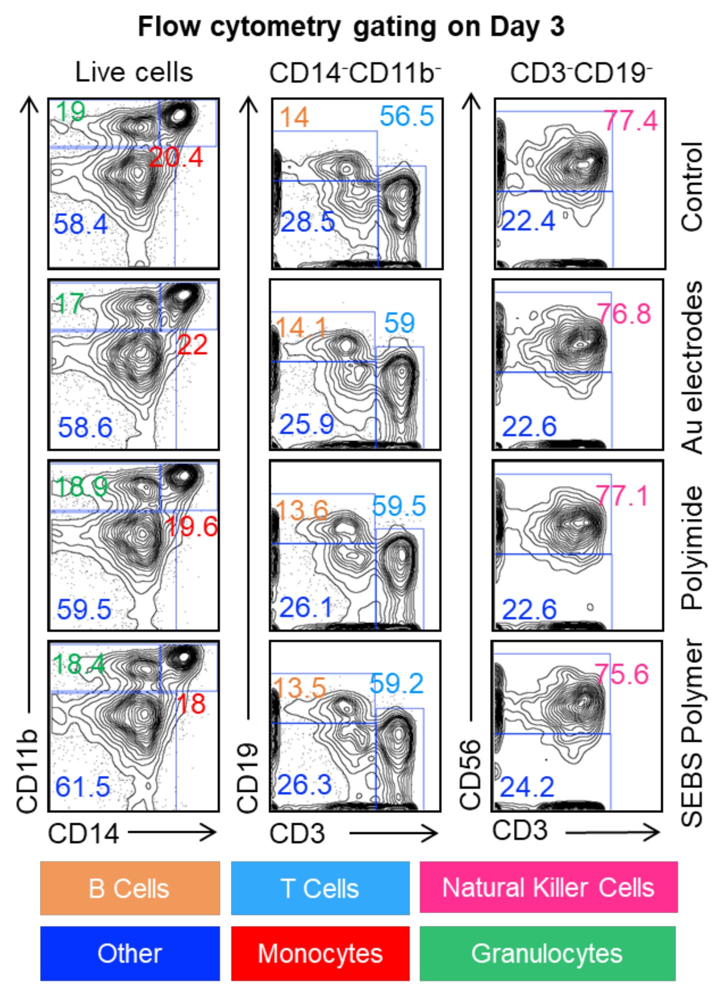

Fig. S10. Representative Fluorescence-Activated Cell Sorting (FACS) analysis. Live cells, including monocytes, granulocytes, T cells, B cells, and natural killer cells, on day 3, is shown. The same gating method was applied to data collected on day 0, day 3, and day 7, enabling monitoring of changes in cell populations over time. This result demonstrates that no discernible changes of major immune cell population (B-cells, T-cells, natural killer cells, monocytes, and granulocytes) in response to the device's materials, and the total cell apoptosis rates remains consistent regardless of the presence or absence of the device's constituent materials, suggesting the device does not induce substantial chronic immune toxicities or cytotoxicity.

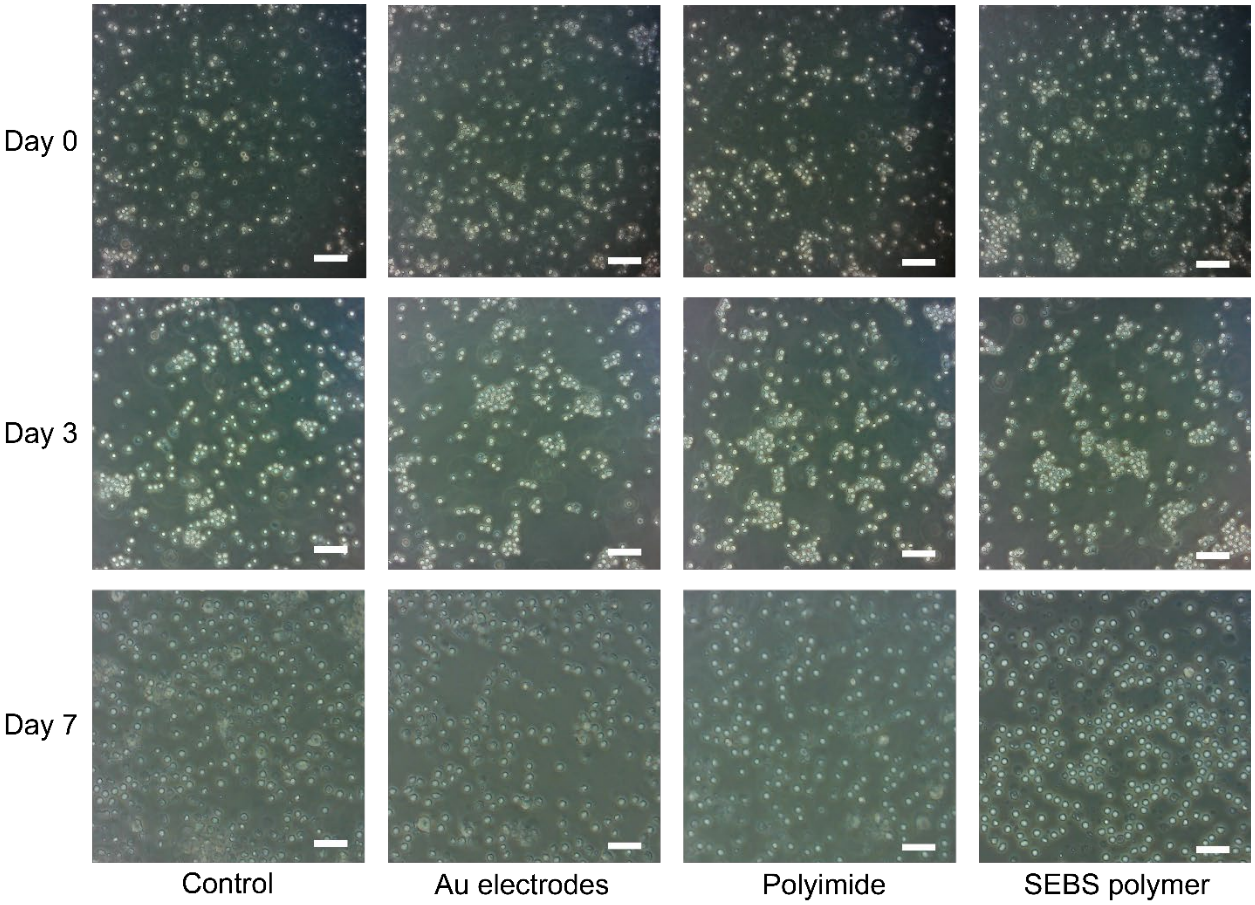

Fig. S11. Microscopic images showing the cell density in biocompatibility test under different condition at varying stages. These images demonstrate that no discernible changes of major immune cell population (B-cells, T-cells, natural killer cells, monocytes, and granulocytes) in response to the device's materials, and the total cell apoptosis rates remains consistent regardless of the presence or absence of the device's constituent materials, suggesting the device does not induce substantial chronic immune toxicities or cytotoxicity. Scale bar: 30μm.

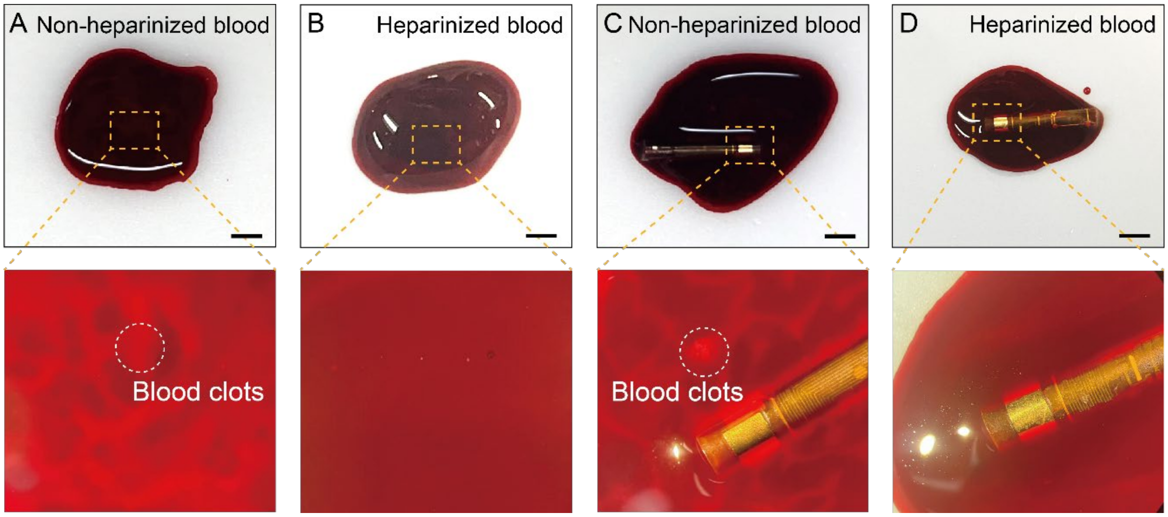

Fig. S12. Heparinized vs. non-heparinized mouse blood and the thrombus vs. no thrombus formation around the microtubular pacemaker. (A) Blood clot formed in the non-heparinized blood after it was extracted from a mouse for 10 minutes. **(B)** Blood clots were absent in the heparinized blood after it was extracted from a mouse for 10 minutes. **(C)** Blood clot formed around the pacemaker in the non-heparinized blood. **(D)** Blood clot formation around the pacemaker was absent in the heparinized blood.

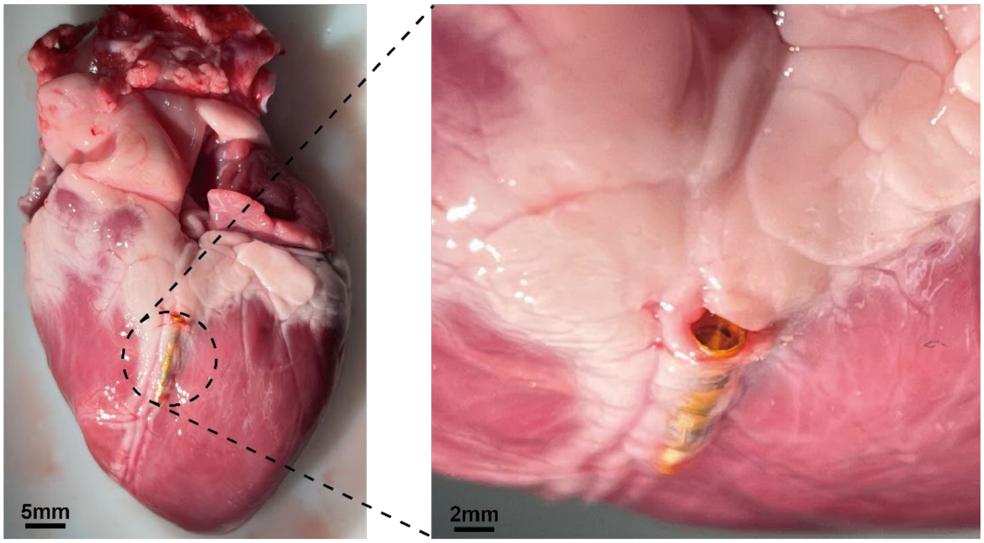

Fig. S13. Photos showing the coronary vein that is chosen for intravascular implantation. The cross-sectional image reveals that the microtubular pacemaker is in close proximity to the vascular wall, establishing an intimate connection. Reasons of choosing this location: (1) its proximity to the bifurcated bundles of left and right ventricles for pacing; (2) its proximity to the chest wall for optimal inductive power transferring.

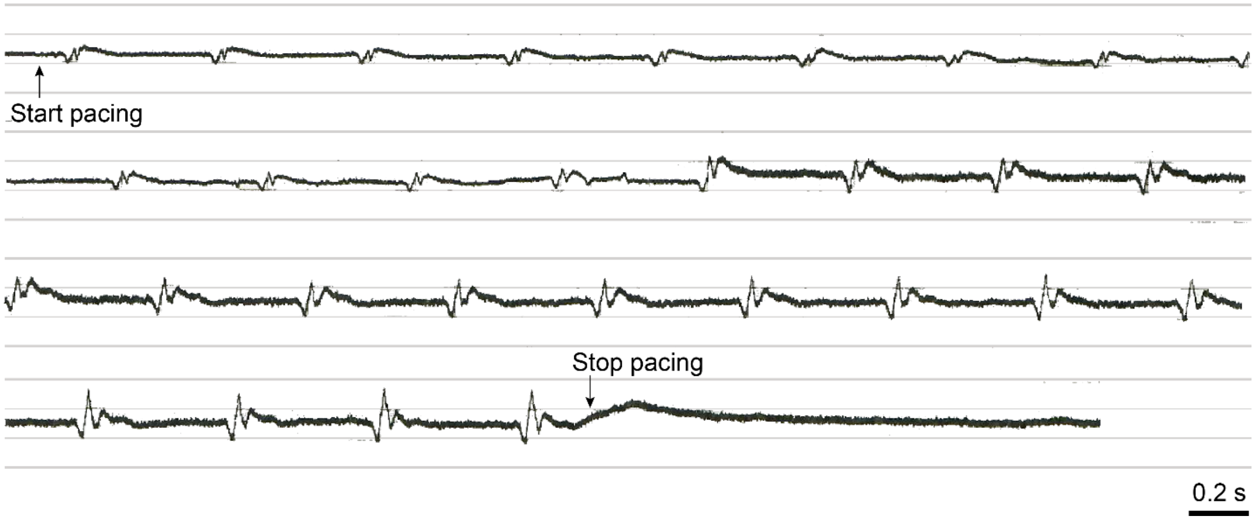

Fig. S14. ECG recording during the active pacing process. The arrows point out when the pacing started and ended.

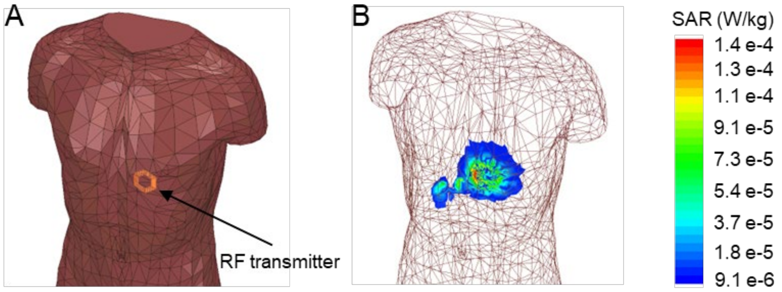

Fig. S15. The simulation results of the specific absorption rate (SAR). (A) A simulated human body model revealing the position of the transmitter. **(B)** The SAR map showing the energy absorption is far below the Federal Drug Administration (FDA)-specified safety level at 10 W/kg.

Movie S1. Recording of the cardiac contraction recovery with an external RF emitter wirelessly transferring the stimulating energy to the microtubular pacemaker.

Movie S2. The real-time monitoring of vital signs and physiological parameters as the pacing rate was increased from 70 bpm to 120 bpm.

Movie S3. Pulsed wave Doppler recording of the femoral arterial blood flow in response to cardiac contraction.

Movie S4. Doppler signals indicating the direction and magnitude of blood velocity toward the hind limb.

Revolutionize cardiac pacing: a biocompatible, battery-free microtubular pacemaker.
